# Psychosomatic symptoms and low psychological well-being in relation to employment status: the influence of social capital in a large cross-sectional study in Sweden

**DOI:** 10.1186/1475-9276-13-22

**Published:** 2014-03-04

**Authors:** Cecilia Åslund, Bengt Starrin, Kent W Nilsson

**Affiliations:** 1 Centre for Clinical Research Västerås, Uppsala University, Västmanland County Hospital Västerås, Västerås, Sweden; 2 Department for Social Studies, Karlstad University, Karlstad, Sweden

**Keywords:** Employment, Public health, Self-rated health, Social capital, Unemployment

## Abstract

**Background:**

Unemployment is associated with adverse effects on health. Social capital has been suggested as a promoter of health via several causal pathways that are associated with the known health risk factors of being unemployed. This cross-sectional study investigated possible additive- and interaction effects of unemployment and five different measures of social capital in relation to psychosomatic symptoms and low psychological well-being.

**Methods:**

A random population sample of 20,538 individuals aged 18–85 years from five counties in Sweden completed a postal survey questionnaire including questions of employment status, psychosomatic symptoms, psychological well-being (General Health Questionnaire-12) and social capital.

**Results:**

Psychosomatic symptoms and reduced psychological well-being were more frequent among unemployed individuals compared with individuals who were employed. Moreover, low social capital and unemployment had additive effects on ill-health. Unemployed individuals with low social capital—specifically with low tangible social support—had increased ill-health compared with unemployed individuals with high social capital. Moreover, to have low social capital within several different areas magnified the negative effects on health. However, no significant interaction effects were found suggesting no moderating effect of social capital in this regard.

**Conclusions:**

Elements of social capital, particularly social support, might be important health-protective factors among individuals who are unemployed.

## Background

Higher levels of psychological ill health are consistently found among unemployed individuals, at all ages and in both sexes [1-3]. In young people, these differences in mental health appear after entry into the labour market showing no differences while still at school [4,5]. Unemployment is moreover associated with physical health problems ranging from physical illness [3] to mortality [6-9] and suicide [10]. Even spouses of unemployed persons suffer a similar increased mortality risk [7]. These patterns are not a general result of those with poor health being at higher risk of unemployment [7], but rather can be explained by four primary factors associated with unemployment: financial stress, social isolation, loss of self-esteem and reduced health-related behaviours [1]. These factors cause psychological and psychosocial stress that through neuroendocrine pathways can be translated into physical manifestations of ill health [11-16]. Indeed, a longitudinal Swedish plant closure study reported consistent increases in cortisol, prolactin, cholesterol, and decreased immune reactions among the unemployed [17,18].

However, the loss of well-being is not uniform. Some people suffer more than others from the loss of a job. Different stress buffering mechanisms may moderate the effect of psychosocial stress on health, protecting people from the pathogenic effects of stress [19]. The concept of social capital is highly interesting in this regard. Social capital is a contextual characteristic that has been defined as the features of social organisation, including for example social and civic participation, collective action and co-operation for mutual benefit [20-23]. It includes a structural and a cognitive component that represent the norms and networks that enable people to take collective action, co-operate and participate socially [24]. The structural component includes societal aspects such as networks, connections and civic participation, whereas the cognitive component includes aspects of trust between individuals, social cohesion and perceived social support [24]. The research field of social capital and health is vast, showing consistent relations to mental ill health [25-27], general ill health [28-30], and mortality [23]. Social capital is suggested as a promoter of health by a number of causal pathways: it helps to decrease psychological and psychosocial stress, provides affective support and acts as a source of self-esteem and mutual respect, and it increases the likelihood that healthy norms of behaviour are adopted [23]. These characteristics of social capital are closely related to the factors that have been suggested to explain the association between unemployment and ill-health; financial stress, social isolation, loss of self-esteem and loss of health-related behaviours [1], indicating that social capital may have a buffering effect on the psychosocial stress of unemployment. A previous study reported that supportive and affiliative relations with wife, friends, and relatives moderated the health consequences of unemployment in men [31]. However, another study investigating the influence of social participation on subjective well-being among unemployed found no moderating effect in relation to unemployment, but strong additive effects [32].

The social capital concept has however been debated, both regarding the definition of the concept and the ways of measuring it in relation to health. For example, it has been measured as social support (i.e., support from family members and friends) [28], generalized trust (i.e., whether the individual felt that other people could generally be trusted) [23,28,33-36], trust in institutions and government (i.e., trust in public servants, government, local councils, large corporations) [34], reciprocity (i.e., whether the individual felt that other people were helpful most of the time) [23,28], social participation (i.e., membership in groups, organizations and voluntary associations) [23,28,29,35,37], neighbourhood cohesion (i.e., neighbourhood integration, connections and safety) [25,34], and voting in national elections [38]. The lack of consistency in the measurements of social capital is a well-known problem within the research field [39]. Studies would therefore benefit from including several different aspects of social capital in relation to health. Thus, the present study will add to the existing literature by both comparing and combining five well used measures of social capital: tangible social support, trust in institutions and government, social participation, neighbourhood cohesion and voting in national elections. These are examined in relation to unemployment and self-reported health in a large representative sample from the general population in Sweden. Moreover, the assumption that there will be an association between unemployment, social capital, and ill-health might generate two specific hypotheses. The direct effect hypothesis suggests that the protecting factor (social capital) influences health regardless of the level of stress an individual experiences, showing an additive effect. Statistically, this would mean that there is no interaction between the protective factor and the stress factor [19]. The buffering hypothesis, on the other hand, suggests that the protective factor moderates the effect of stress on health. The existence of such an effect requires a statistically significant interaction between the protective factor and the stress factor [19]. The present study aims to investigate the possible additive- and interaction effects of unemployment and low social capital in relation to psychosomatic symptoms and low psychological well-being.

## Methods

A postal survey was distributed in the five Swedish counties of Uppsala, Sörmland, Västmanland, Värmland, and Örebro, together comprising approximately 1,400,000 inhabitants, during March to May 2008. A random sample of 68,710 individuals, aged 18–84 years and stratified by sex, age, and city (and parts of the city for larger cities), were drawn from the total population by Statistics Sweden and asked to participate in the present study via a postal survey questionnaire supplied with prepaid return envelopes. The only inclusion criterion was being registered as a Swedish citizen. There were no exclusion criteria. After 10 days, a reminder was sent to the participants who had not responded. Ten days after the first reminder, a new questionnaire was sent together with a second reminder. A new questionnaire together with a third reminder was sent four weeks after the first reminder. The questionnaires were scanned and transformed into a data file with no personal identification of the participants.

A total of 40,674 individuals responded (59.2% response rate), of which 18,499 (45.5%) were men and 22,175 (54.5%) were women. The mean age was 53.8 years (standard deviation, SD =17.9). The educational level among the participants was compulsory school 21.3%, upper secondary school 40.8%, college or university ≤2 years, 4.4%, and college or university >2 years, 21.1%. Self-reported educational information was missing in 12.3% of the participants. The response rates were 54.5–62.2% between the five counties and differed depending on sex (men, 51.4–53.7%; women, 63.2–66.7%), age (18–34 years old, 41.2–48.5%; 35–49 years old, 51.1–55.7%; 50–64 years old, 66.1–68.6%; 65–79 years old 72.4–79.5%; 80–84 years old, 60.8–74.7%), education (compulsory school, 52.5–54.3%; upper secondary school, 55.6–57.9%; college or university ≤2 years, 57.9–61.9%; college or university >2 years, 65.6–68.1%), ethnicity (Swedish-born individuals, 58.6–62.5%; participants from other Nordic countries, 56.9–62.9%; participants from other non-Nordic countries, 40.9–43.8%), and employment status (employed, 57.2–61.2%; unemployed, 43.9–49.1%; students, 47.0–52.9%; others, 59.4–64.4%). Out of this total population, 20,136 individuals who were neither employed nor unemployed (students; on leave/parental leave/sick leave; housewife/husband; retired, or on other maintenance) were excluded from this study, leaving 20,538 participants.

The study followed the Swedish guidelines for studies of social sciences and humanities according to the Declaration of Helsinki. According to Swedish regulations, this type of study no longer applies for ethical approval by a medical faculty.

### Measures of demographic data

#### ***Demographic background***

Sex was categorized as (1) men and (2) women. Level of education was categorized as (1) compulsory school, (2) upper secondary school, (3) college or university ≤2 years, (4) college or university >2 years. Age was categorized as (1) 18–34 years old; (2) 35–49 years old; (3) 50–64 years old; (4) 65–79 years old; (5) 80–84 years old.

#### ***Employment***

Employment was categorized as 1, employed (work as an employee or self-employed); 2, unemployed.

### Measures of social capital

#### ***Tangible social support***

This was measured by the following questions. Do you have persons around you who would give you support in the event of personal problems or crises? Do you have persons around you who would help you with grocery shopping/cooking if you should turn ill? Do you have persons around you who would help you if you were to move to another dwelling? Answer alternatives were: 1, no; 2, probably not; 3, yes, probably; and 4, yes, definitely. The internal consistency of the questions of tangible social support was α = 0.773. A summation index was created with a range of 4–12 points. The index was divided into quartiles (Qs) and dichotomized where Q2–Q4 (>25%) counted as high tangible social support (0) and Q1 (<25%) counted as low tangible social support (1). This measure was adapted according to previous measurements of tangible or instrumental social support [40,41].

#### ***Member of an organization***

This was tested by the following questions. Do you partake in activities or go to meetings in any group, organization, community, or society? Answer alternatives were: no; yes, labour union; yes, political party; yes, nature or environmental organization; yes, sports association; yes, aid organization; yes, disability or patient’s association; yes, senior citizens society; yes, religious organization or church; yes, choir, orchestra, theatre or other cultural group; yes, housing society; yes, parent association or similar; yes, old homestead community; yes, other. A summation index was created for the number of memberships in organizations with a range of 0–13 points. A division of the index by quartiles was not optimal due to the distribution (58.6% were non-members, 29.0% were members in one organization and 12.3% were members in two or more organizations). A dichotomized variable was therefore created where participants who answered at least one “yes” on the above questions were classified as member of an organization (0) and the remaining participants were classified as not member of an organization (1). This measurement was an adaption of the Kawachi [42] measure of membership in voluntary groups, also as suggested by others [40].

#### ***Neighbourhood social trust***

Neighbourhood social trust was measured by responding to four statements about the participant’s neighbourhood as follows. People in this neighbourhood can be trusted. One can feel safe and secure from assault or threat in this neighbourhood. The people in this neighbourhood know each other well. The people in this neighbourhood care about each other. Answer alternatives were: 1, completely true; 2, rather true; 3, rather false; or 4, completely false. The internal consistency of the questions of neighbourhood social trust was α = 0.819. A summation index was created with a range of 4–16 points. The index was divided by quartiles and dichotomized where Q2–Q4 (>25%) counted as high neighbourhood social trust (0) and Q1 (<25%) counted as low neighbourhood social trust (1). This measurement was an adaption of the collective efficacy scale (social cohesion and trust) developed by Sampson et al [43].

#### ***Trust in societal institutions***

Trust in societal or governmental institutions is a commonly used social capital measurement [40,41,44]. The respondents were asked to rate their trust in: health care, school, child care, care of senior citizens; the Human Service Agency; the Social Insurance Association; the employment service; the unions; the police; the law courts; the politicians in the community council; the politicians in the county council; the politicians in the parliament; or the politicians in the government. The rate alternatives were: 1, very high; 2; rather high; 3; not particularly high; or 4, none. The internal consistency of the questions of trust in societal institutions was α = 0.890. A summation index was created with a range of 14–56 points. The index was divided by quartiles and dichotomized where Q2–Q4 (>25%) counted as high trust in societal institutions (0) and Q1 (<25%) counted as low trust in societal institutions (1).

#### ***Voting in the last general election***

The participants were asked whether they voted in the last general election for the Swedish parliament. Answers were 1, yes or 2, no. Only individuals who were eligible for voting at the time were assigned a value. A total of 1 379 individuals (6.7%) were not eligible for voting in the last general election. This measure of civic participation is a commonly used social capital measurement [40,41].

### Measures of health

#### ***Psychosomatic symptoms***

This section included the following questions. How often during the last three months have you experienced the following symptoms: (i) pain in the shoulders/neck; (ii) pain in the back/hips; (iii) pain in the hands/arms/legs/knees/feet; (iv) abdominal pain; (v) Headache/migraine; (vi) Anxiety/nervousness; (vii) Feelings of fatigue/feebleness; (viii) sleeping problems; (ix) depression; (x) dizziness; (xi) irritated mucous membranes; (xii) stress? Answer alternatives were: 0, never; 1, rarely; 2, several times; 3, most of the time. The internal consistency of the questions of psychosomatic symptoms was α = 0.860. A summation index was created with a range of 0–36 points. The index was divided by standard deviations (SDs) and dichotomized with +1 SD as the cut-off point for having many psychosomatic symptoms.

#### ***Psychological well-being***

The short version of the General Health Questionnaire (GHQ-12) was used. In this study, Goldberg’s GHQ scoring method was applied [45]. The responders can score 0–0–1–1 giving a total sum of 0 – 12 points. The internal consistency of the GHQ-12 items was α = 0.895. A score of ≥3 was categorized as reduced psychological well-being.

#### ***Other chronic disease***

The participants were asked whether they suffered from any long-lasting disease (> 6 months), any persisting symptoms following an accident, any disability, or any other long-lasting health problem. Answer alternatives were: 1, no; 2, yes.

### Statistical analyses

Sex differences in the demographic factors were analysed by χ^2^ tests. Differences in psychosomatic symptoms, psychological well-being and the social capital variables among individuals who were employed compared with unemployed were analysed using the Mann–Whitney nonparametric *U* test. The additive associations of employment status and social capital in relation to psychosomatic symptoms and psychological well-being were analysed in two ways. First, we created four quadrant models of employment status and the separate social capital variables by combining the two levels of employment status (employed/unemployed) with the two levels of each social capital variable (high/low tangible social support, member/not member of an organization, high/low neighbourhood social trust, high/low trust in societal institutions, voted/did not vote in the last general election). The four quadrant models were analysed in five separate univariate categorical binary logistic regressions adjusted for age, sex, educational level, and chronic disease to investigate any associations with psychosomatic symptoms and psychological well-being. Second, we summed the five dichotomized social capital variables into one index, where low tangible social support, no membership in any organization, low neighbourhood social trust, low trust in societal institutions and non-voting in the last general election each generated 1 point (range 0–5 points). The index was further adjusted to compensate for the small number in some of the groups: 0 points (0); 1 point (1); 2 points (2); 3 points (3), 4–5 points (4). The summated social capital variable and the variable for employment status were inserted in a multivariate categorical binary logistic regression analysis, adjusted for age, sex, educational level, and chronic disease to investigate the association between psychosomatic symptoms and psychological well-being. Furthermore, interaction effects of employment status in relation to each of the dichotomized social capital variables were analysed by binary logistic regressions. All statistical analyses were performed using SPSS (Statistical Package for Social Sciences 20.0) for Windows.

## Results

The group of individuals who were excluded from the study because they were neither employed nor unemployed reported lower tangible social support (*M* = 10.92, SD = 1.66, *Z* = –20.37, *p* < 0.001), lower neighbourhood social capital (*M* = 12.87, SD = 2.38, *Z* = –5.21, *p* < 0.001) and lower trust in societal institutions (*M* = 25.11, SD = 10.79, *Z* = –30.66, *p* < 0.001) compared with the included study participants. A higher proportion of the excluded individuals reported being member of at least one organization (44.5%, *Z* = –6.28, *p* < 0.001), but there was no difference with regards to voting in the last general election (93.1%, *Z* = –1.82, *p* = 0.069). Moreover, the excluded individuals had a higher rate of psychosomatic symptoms (*M* = 9.07, SD = 6.69, *Z* = –12.43, *p* < 0.001), but there was no difference in psychological well-being (*M* = 1.15, SD = 2.52, *Z* = –0.11, *p* = 0.915) compared with the included study participants. Missing data on the key variables ranged between 0.0% - 8.1% (the last number regards voting in the last general election), with a medium internal non-response rate of 1.3%.

Descriptions of the study participants are presented in Table 1. Mean values for the social capital measures were as follows. The tangible social support index *M* = 11.23, SD = 1.38 (Men, *M* = 11.10, SD = 1.49; Women, *M* = 11.35, SD = 1.26, *Z* = –14.52, *p* < 0.001); the neighbourhood social capital index *M* = 12.78, SD = 2.28 (Men, *M* = 12.88, SD = 2.24; Women, *M* = 12.70, SD = 2.32, *Z* = –5.62, *p* < 0.001); the trust in societal institutions index *M* = 28.42, SD = 9.70 (Men, *M* = 28.89, SD = 9.36; Women, *M* = 28.00, SD = 9.98, *Z* = –6.01, *p* < 0.001). A total of 8358 (41.4%) reported being member of at least one organization (43.8% of men and 39.1% of women; *Z* = –6.82, *p* < 0.001) and among the participants that were eligible for voting a total of 17,662 (93.6%) voted in the last general election (93.3% of men and 93.8% of women; *Z* = –1.51, *p* = 0.131). The mean value of the psychosomatic symptoms index was *M* = 8.54, SD = 6.28 (Men, *M* = 6.92, SD = 5.59; Women, *M* = 9.89, SD = 6.50, *Z* = –48.54, *p* < 0.001). The mean value of the psychological well-being index was *M* = 1.14, SD = 2.47 (Men, *M* = 0.85, SD = 2.13; Women, *M* = 1.38, SD = 2.70, *Z* = –22.77, *p* < 0.001).

**Table 1:** Description of the study participants

	**Total**		**Men**		**Women**			
	***n***	**%**	***n***	**%**	***n***	**%**	**χ**^**2**^	***p***
**Sex**	20538	100	9734	47.4	10804	52.6	55.75	<0.001
**Age in years**								
18–34	4812	23.4	2164	45.0	2648	55.0		
35–49	7073	34.4	3233	45.7	3840	54.3		
50–64	7969	38.8	3840	48.2	4129	51.8		
65–79	661	3.2	482	72.9	179	27.1		
80–84	23	0.1	15	65.2	8	34.8		
Total	20538	100					197.07	<0.001
**Educational background**								
Compulsory school	2902	14.3	1704	58.7	1198	41.3		
Upper secondary school	10393	51.1	5048	48.6	5345	51.4		
College or university <2 years	1142	5.6	682	59.7	460	40.3		
College or university >2 years	5900	29.0	2188	37.1	3712	62.9		
Total	20337	100					476.16	<0.001
**Other chronic disease**								
No	15794	78.0	7598	48.1	8196	51.9		
Yes	4456	22.0	2020	45.3	2436	54.7		
Total	20250	100					10.73	0.001
**Employment status**								
Employed	19528	95.1	9347	47.9	10181	52.1		
Unemployed	1010	4.9	387	38.3	623	61.7		
Total	20538	100					35.11	<0.001

### Employment status, social capital and health

Individuals who were unemployed had more psychosomatic symptoms and reduced psychological well-being compared with individuals who were employed (Table 2). Moreover, unemployed individuals had lower tangible social support, were less often a member of an organization, had lower neighbourhood social trust, had lower trust in societal institutions and were less likely to have voted in the last general election (Table 2).

**Table 2:** **Means, medians, SDs, *Q1–Q4 *** **and mean rank on psychosomatic symptoms, reduced psychological well-being, social support, number of memberships in organizations, neighbourhood social trust, trust in societal institutions and voting in the last general election among employed and unemployed**

	***n***	**Mean**	**Median**	**SD**	***Q1–Q4***	**Mean rank**^**a**^	***Z***	***p***
*Psychosomatic symptoms*								
Employed	19477	7.91	7.00	5.72	3.00–11.00	10122		
Unemployed	1005	10.76	10.00	7.02	5.00–15.00	12547		
							–12.70	<0.001
*Reduced psychological well-being*								
Employed	19471	1.05	0.00	2.34	0.00–1.00	10105		
Unemployed	1004	2.57	1.00	3.63	0.00–4.00	12821		
							–17.87	<0.001
*Social support*								
Employed	19370	11.26	12.00	1.33	11.00–12.00	10258		
Unemployed	996	10.67	12.00	1.99	10.00–12.00	8725		
							–9.42	<0.001
*Number of memberships in organizations*								
Employed	19210	0.59	0.00	0.83	0.00–1.00	10178		
Unemployed	997	0.36	0.00	0.68	0.00–1.00	8680		
							–8.99	<0.001
*Neighbourhood social trust*								
Employed	19415	12.83	13.00	2.25	12.00–14.00	10317		
Unemployed	1001	11.85	12.00	2.63	10.00–14.00	8096		
							–11.76	<0.001
*Trust in societal institutions*								
Employed	18956	28.53	29.00	9.64	22.00–35.00	10030		
Unemployed	967	26.32	26.00	10.50	18.00–33.00	8634		
							–7.36	<0.001
*Voted last general election* (Yes =1, No =2)								
Employed	18058	1.06	1.00	0.24	1.00–1.00	9405		
Unemployed	819	1.14	1.00	0.35	1.00–1.00	10180		
							–9.34	<0.001

^a^Mann–Whitney *U*-test.

The largest health effect of the social capital factors was found for tangible social support, where individuals with low tangible social support had more psychosomatic symptoms and reduced psychological well-being compared with individuals with high tangible social support, regardless of employment status (Table 3). However, the effect of the social capital factors was particularly evident among unemployed individuals. For such subjects with low tangible social support, about one out of two reported many psychosomatic symptoms and reduced psychological well-being. Moreover, these individuals had approximately four times higher odds for psychosomatic symptoms and six times higher odds for reduced psychological well-being compared with individuals who were employed and had high tangible social support. Among unemployed individuals who were not member of any organization, had low neighbourhood social trust, had low trust in societal institutions or who did not vote in the last general election, about four out of ten had many psychosomatic symptoms and reduced psychological well-being. Moreover, these individuals had approximately two- to three-fold increased odds for psychosomatic symptoms and reduced psychological well-being compared with individuals who were employed and had high social capital within these factors (Table 3). However, in separate binary logistic regression analyses testing for the interaction between employment status and each of the social capital variables, no significant interactions were found (data not shown).

**Table 3:** Univariate categorical binary logistic regressions of having many psychosomatic symptoms and reduced psychological well-being among employed and unemployed, in combination with high vs. low social support, being a member of an organization or not, having high vs. low neighbourhood social trust, having high vs. low trust in societal institutions and voting in the last general election or not

	**Many psychosomatic symptoms**					**Reduced psychological well-being**				
	**%**	***n***	***OR***^***a***^	***95% CI***	***p***	**%**	***n***	***OR***^***a***^	***95% CI***	***p***
*Employed – high social support*	17.5	2680	1 (ref)			12.4	1901	1 (ref)		
Employed – low social support	30.7	1096	2.281	2.087–2.494	<0.001	23.8	849	2.508	2.280–2.758	<0.001
Unemployed – high social support	32.0	210	1.605	1.337–1.927	<0.001	26.7	175	2.030	1.683–2.450	<0.001
Unemployed – low social support	52.2	154	4.275	3.321–5.502	<0.001	48.0	142	6.127	4.801–7.819	<0.001
*Employed – member of an organization*	17.6	1388	1 (ref)			14.0	1105	1 (ref)		
Employed – not member of an organization	21.7	2353	1.291	1.193–1.397	<0.001	15.0	1625	1.089	1.000–1.186	0.051
Unemployed – member of an organization	35.2	95	1.994	1.514–2.627	<0.001	28.4	77	2.113	1.596–2.796	<0.001
Unemployed – not member of an organization	38.8	264	2.206	1.843–2.640	<0.001	34.8	236	2.638	2.203–3.158	<0.001
*Employed – high neighbourhood social trust*	17.3	2494	1 (ref)			12.3	1767	1 (ref)		
Employed – low neighbourhood social trust	28.4	1292	1.801	1.657–1.957	<0.001	21.7	990	1.730	1.582–1.890	<0.001
Unemployed – high neighbourhood social trust	34.0	198	1.900	1.570–2.300	<0.001	29.3	171	2.512	2.073–3.045	<0.001
Unemployed – low neighbourhood social trust	44.6	166	2.753	2.198–3.447	<0.001	39.4	146	3.366	2.695–4.205	<0.001
*Employed – high trust in societal institutions*	19.2	2886	1 (ref)			14.1	2120	1 (ref)		
Employed – low trust in societal institutions	23.9	825	1.222	1.112–1.343	<0.001	16.9	581	1.256	1.132–1.394	<0.001
Unemployed – high trust in societal institutions	35.5	234	1.693	1.417–2.024	<0.001	30.8	203	2.201	1.839–2.633	<0.001
Unemployed – low trust in societal institutions	44.5	118	2.512	1.929–3.271	<0.001	40.0	106	3.466	2.671–4.498	<0.001
*Employed – voted in last general election*	19.6	3252	1 (ref)			14.2	2359	1 (ref)		
Employed – did not vote last general election	23.0	246	1.142	0.975–1.338	0.087	15.9	170	1.090	0.915–1.299	0.335
Unemployed – voted in last general election	37.6	258	1.824	1.535–2.168	<0.001	32.1	221	2.352	1.977–2.797	<0.001
Unemployed – did not vote in last general election	44.6	50	2.620	1.754–3.913	<0.001	38.4	43	3.079	2.065–4.590	<0.001

Presenting percentages, numbers, odds ratio (OR), 95% confidence interval (CI) and p values, adjusted for age, sex, educational level, and chronic disease.

### Additive effect of the social capital measures

We further explored the effect of low social capital by summarizing the number of factors where an individual had low social capital into one variable. In a multivariate analysis including the summarized social capital variable, unemployment accounted for 60% increased odds for many psychosomatic symptoms and two-fold increased odds for reduced psychological well-being (Table 4). The odds for psychosomatic symptoms and reduced psychological well-being increased with the number of present low social capital factors, where four or more low social capital items were associated with approximately four-fold increased odds (Table 4).

**Table 4:** Multivariate categorical binary logistic regression analysing the effect of the summation of number of low social capital variables

	**Many psychosomatic symptoms**			**Reduced psychological well-being**		
	**OR**^**a**^	**95% CI**	***p***	**OR**^**b**^	**95% CI**	***p***
*Employed*	1 (ref)			1 (ref)		
Unemployed	1.622	1.372–1.918	<0.001	2.113	1.787–2.500	<0.001
*0 low social capital items*	1 (ref)			1 (ref)		
1 low social capital item	1.378	1.233–1.540	<0.001	1.207	1.070–1.362	0.002
2 low social capital items	1.982	1.762–2.229	<0.001	1.643	1.447–1.867	<0.001
3 low social capital items	2.873	2.478–3.330	<0.001	2.612	2.235–3.052	<0.001
4–5 low social capital items	4.371	3.432–5.569	<0.001	3.906	3.050–5.002	<0.001

Low social support, no organization membership, low neighbourhood social trust, low trust in societal institutions and non-voting in the last general election each counted as one low social capital item. Adjusted for sex, age, educational level, and chronic disease.

^a^Nagelkerke’s R^2^ = 0.181.

^b^Nagelkerke’s R^2^ = 0.108.

Individuals aged 65–84 years would normally be classified as retired in Sweden, although a total of 684 participants in this age group stated that they were employed or unemployed in the questionnaire (Table 1). Therefore, all analyses were rerun with the exclusion of the participants in this age group. This procedure did not significantly alter the results in any way.

## Discussion

The present study aimed at investigating the possible additive- and interaction effects of unemployment and five different measures of social capital in relation to psychosomatic symptoms and low psychological well-being. The main findings were: 1) psychosomatic symptoms and reduced psychological well-being were more frequent among unemployed individuals compared with individuals who were employed; 2) unemployed individuals reported lower levels of social capital than employed individuals; 3) low social capital and unemployment had additive effects on ill-health, where unemployed individuals with low social capital—specifically low tangible social support—had increased health problems compared with unemployed individuals with high social capital; 4) having low social capital within several different social capital areas multiplied the odds of ill health. However, no interactions between social capital and unemployment were found, suggesting no moderating or buffering effect of social capital in this regard.

Social capital has been suggested as a mediating factor in the associations between income inequality and ill health in industrialized countries [42], an association that is stronger in countries with large income inequalities [46]. Low socioeconomic status and income inequality might result in less trusting and reciprocal relationships between people and lower levels of civic and political participation [15,34,47,48]. However, even in more egalitarian countries such as Sweden there is an association between social capital and health. Social capital has been suggested as a promoter of health by a number of causal pathways that includes several of the known risk factors associated with unemployment [23]. In the present study, the association between low social capital and ill health was particularly evident among unemployed, a group at high risk of ill health because of increased financial stress, social isolation, loss of self-esteem and reduced health promoting behaviours [1]. Unemployment might not only bring exclusion from work, but also exclusion from the social capital, and this additive effect might be part of the explanation for the association between unemployment and ill health.

However, although we found additive effects of low social capital and unemployment on ill-health, no interaction or moderating effects of social capital were found. A previous study investigating the influence of social participation on subjective well-being among unemployed reported similar findings with a strong association between high social capital and well-being but no moderating effect in relation to unemployment [32]. In contrast, another study claimed that supportive and affiliative relations with wife, friends, and relatives moderated the health consequences of unemployment in men [31]. However, that study did not test for interaction effects. The buffering hypothesis suggests that a specific factor buffers or moderates the effect of stress on health, protecting people from the pathogenic effects of the stressor [19]. The existence of such an effect requires a statistically significant interaction between the buffering factor and the stressor [19]. Regarding the buffering hypothesis of social support, it has been further suggested that the buffering effect for a specific life stress will only be observed for social support aimed at alleviating the specific stress [49]. It seems plausible that this suggestion might be transmittable to social capital and could explain the lack of interaction effects of social capital in the present study. Although the social capital measures had additive effects on health, the measures might not be sufficiently related to the stressors associated with unemployment to show moderating effects on the individual level.

Concerning the additive effect of social capital, it seems plausible that the consequences of losing a job and entering unemployment might by the very nature of the situation involve decreased social capital. The psychosocial stress of losing one’s job might also cause an increase in ill health, which is further accentuated by the loss of social capital with less means to cope with the stress through—for example—social participation and support. This additive effect of unemployment and low social capital suggests that efforts to maintain or even increase the individual’s social capital could increase the chances of maintaining psychosocial health during unemployment.

The present study has several limitations. First, the cross-sectional design excluded any possibility for analysing directions of cause and effect. Although the results show strong associations between social capital, unemployment, and ill health, there is always a major risk of reverse causation. Low psychological well-being and psychosomatic symptoms might for example be related to higher rates of sick leave and difficulties in finding and keeping a job. Moreover, general ill-health might result in a less active leisure time and a diminished social life which substantially could decrease the social capital. Second, the total response rate was not optimal (59.2%) and the response rate differed between groups. For example, the response rate was lower among men within the younger age groups, those with lower education, those from non-Nordic countries and those who were unemployed. This might have affected our results, specifically regarding the analyses of ill health in relation to employment status. The aim of the study was to investigate the associations between the different measures of social capital and ill health, specifically in relation to employment status. Therefore, individuals who were neither employed nor unemployed (e.g., those who were studying, on leave/parental leave/sick leave, living as a housewife/husband, retired, or on other maintenance) were excluded from the study. However, as these individuals generally reported lower social capital and more pronounced health problems, the inclusion of them would probably have yielded even stronger associations between social capital and ill health. Third, the measurements of social capital were not validated. To our knowledge, there is no validated gold standard for the measurement of social capital. We have however considered the content validity, face validity, and construct validity of the measurements as suggested by Harpham et al [50]. Regarding the measure of tangible social support, it did not specifically ask about support in relation to the hazards of unemployment, which is a limitation. However, the types of assistance described (whether the person could expect aid in personal crises, get help with daily chores if they got sick, get help if they were moving) may still be crucial to the easing or solving of closely related stress factors. Fourth, social capital and ill health could moreover be related to several confounding demographic and psychosocial factors that were not controlled for in the present study and that might partly explain the relationships between social capital and ill health, i.e., occupational class, income, marital status, long-term unemployment, and trade conditions in society. For example, it has been shown that the association between unemployment and mortality weakens as the general unemployment rate increases [51]. Fifth, we chose to dichotomize the social capital variables by using the cut-off of quartiles in order to identify the 25% of the participants with the lowest social capital scores within each variable. There are several different ways to perform statistical cut-offs for dichotomization, i e medians, quartiles, and SD:s. We found the quartiles cut-off to be the most convenient in relation to our aim and the statistical analyses. To split the population by the median would be less compatible with our aim, since the normal distribution of the population would mean that the majority of the individuals classified to the “high” and “low” social capital groups in truth would be closer to “medium” social capital.

The limitations of this study could be balanced by the statistical power. There were 20,538 respondents from the general population of five different counties in Sweden. Another strength is that we were able to compare and combine five different measures of social capital; tangible social support, trust in institutions and government, social participation, neighbourhood cohesion and voting in national elections. Although these five measures of social capital are well known and often used within the research field, most previous studies have focused on one or two of them. The combination of five aspects of the concept of social capital in one study is not common and contributes important information to this research field.

## Conclusions

Social capital, particularly tangible social support, may be an important health protective factor among individuals who are unemployed. Unemployment involves several factors related to stress and ill health that also have associations to social capital. Efforts to maintain and increase their individual social capital may increase the chances of maintained psychosocial health among the unemployed. Future studies are however needed to further investigate the protective effect of social capital on health, particularly among individuals under psychosocial stress such as unemployment or financial strain. Moreover, future research would benefit from investigating different aspects of the social capital concept in relation to health, specifically possible additive effects.

## Abbreviations

GHQ-12: General health questionnaire.

## Competing interests

The authors declare that they have no competing interest.

## Authors’ contributions

Study concept and design: CÅ, BS, KWN. Acquisition of data: BS. Questionnaire management: BS. Analysis and interpretation of data: CÅ, KWN. Drafting of manuscript: CÅ, KWN. Critical revision: CÅ, BS, KWN. All authors read and approved the final manuscript.
